# Assessing the Role of Medical Caption Technology to Support Physician-Patient Communication for Patients With Hearing Loss: Mixed Methods Pilot Study

**DOI:** 10.2196/79073

**Published:** 2026-01-15

**Authors:** Sarah E Hughes, Liang-Yuan Wu, Lindsay J Ma, Dhruv Jain, Michael M McKee

**Affiliations:** 1 University of Michigan Medical School, Ann Arbor, MI, United States; 2 Department of Computer Science and Engineering, University of Michigan, Ann Arbor, MI, United States; 3 Department of Family Medicine, University of Michigan, 1018 Fuller St., Ann Arbor, MI, 48104-1213, United States, 17345395000, 17344264370; 4 Department of Physical Medicine and Rehabilitation, University of Michigan Medical School, Ann Arbor, MI, United States

**Keywords:** health communication, hearing loss, deafness, speech recognition software, usability testing, health care accessibility

## Abstract

**Background:**

Speech recognition technology is widely used by individuals who are Deaf/deaf and hard-of-hearing (DHH) in everyday communication, but its clinical applications remain underexplored. Communication barriers in health care can compromise safety, understanding, and autonomy for individuals who are DHH.

**Objective:**

This study aimed to evaluate a real-time speech recognition system (SRS) tailored for clinical settings, examining its usability, perceived effectiveness, and transcription accuracy among users who are DHH.

**Methods:**

We conducted a pilot study with 10 adults who are DHH participating in mock outpatient encounters using a custom SRS powered by Google’s speech-to-text application programming interface. We used a convergent parallel mixed-methods design, collecting quantitative usability ratings and qualitative interview data during the same study session. These datasets were subsequently merged and jointly interpreted. Participants completed postscenario surveys and structured exit interviews assessing distraction, trust, ease of use, satisfaction, and emotional response. Caption accuracy was benchmarked against professional communication access real-time translation transcripts using word error rate (WER). Because WER assigns equal weight to all tokens, it does not differentiate between routine transcription errors and those involving safety-critical clinical terms (eg, medications or diagnoses). Therefore, WER may underestimate the potential impact of certain errors in medical contexts.

**Results:**

Across 29 clinical scenario simulations, 86% (25/29) of participants found captions nondistracting, 90% (26/29) reported them easy to follow and trustworthy, and 76% (22/29) were satisfied with the experience. Participants described the SRS as intuitive, emotionally grounding, and preferable to lip reading in masked settings. WER ranged from 12.7% to 22.8%, consistent with benchmarks for automated SRSs. Interviews revealed themes of increased confidence in following clinical conversations and staying engaged despite masked communication. Participants reported less anxiety about missing critical medical information and expressed a strong interest in expanding the tool to real-world settings, especially for older adults or those with cognitive impairments.

**Conclusions:**

Our findings support the potential of real-time captioning to enhance accessibility and reduce the cognitive and mental burden of communication for individuals who are DHH in clinical care. Participants described the SRS as both functionally effective and personally empowering. While accuracy for complex medical terminology remains a limitation, participants consistently expressed trust in the system and a desire for its integration into clinical care. Future research should explore real-world implementation, domain-specific optimization, and the development of user-centered evaluation metrics that extend beyond transcription fidelity to include trust, autonomy, and communication equity.

## Introduction

Effective communication is foundational to safe, equitable, and high-quality health care [1]. However, individuals who are Deaf/deaf and hard-of-hearing (DHH) often face communication barriers that compromise understanding and autonomy [2]. These barriers contribute to poor health outcomes and reduced patient engagement in real-time clinical settings [2]. The scale of this issue highlights the need to understand which communication support tools are available and provided, and to whom. In the United States, an estimated 48 million people live with some degree of hearing loss (HL), and 1 in 3 adults older than 65 years experiences disabling age-related hearing loss [3,4]. Despite this growing population, access to communication supports remains inconsistent [5,6].

Deaf individuals who use American Sign Language often receive interpreter services [7]. In contrast, oral communicators with people with HL who normally rely on spoken English are less likely to receive accommodations such as captioning, assistive listening devices, or environmental modifications [7]. Especially in clinical workflows, interpreter services are systematically implemented, whereas accommodations for oral communicators are likely not [8-12]. This gap persists despite longstanding mandates under the Americans with Disabilities Act, which mandates effective communication in health care [13]. As a result, many patients who are DHH still receive incomplete or delayed health information [5]. These gaps undermine informed decision-making, autonomy, and overall care outcomes [14,15]. Far from logistical oversights, these structural inequities perpetuate persistent disparities in care for individuals who are DHH.

These long-standing disparities became even more visible during the COVID-19 pandemic [14]. Universal masking eliminated lip reading and facial cues, which were essential supports for many individuals who are DHH and rely on oral communication [16]. This shift underscored the need for scalable solutions to maintain accessible communication in high-stakes settings [14,17].

Real-time captioning is 1 solution for improving communication access for individuals who are DHH when traditional strategies (eg, lip reading or interpreters) are unavailable [18]. Captioning tools can be deployed quickly and readily support both in-person and virtual communication [19]. However, captioning accuracy of clinical conversations may be affected by terminology unique to the medical field or speaker attribution and is understudied [19,20]. This has left a critical gap in the development of effective and equitable access tools.

By allowing both conversation partners to see each other’s faces while reading the same captions, transparent or dual-visibility captioning preserves the natural flow of spoken interaction and is a promising solution for clinical communication. Prior work, such as See-Through Captions [21], See-Through Captions in a Museum Guided Tour [22], and Wearable Subtitles [23], has primarily focused on general or educational settings. Our study extends this line of research into medical contexts, where communication accuracy can directly affect patient safety and outcomes. It also emphasizes the emotional and psychological impact of captioning during clinical interactions and addresses the unique technical challenges posed by medical vocabulary and workflow integration.

In summary, we developed and evaluated a real-time captioning tool using Google’s speech-to-text engine to generate live captions during simulated clinical encounters. We tested this system in dynamic, medically relevant scenarios designed to simulate typical ambulatory care encounters. In this pilot study, we explored how individuals who are DHH experienced the captioning system in these simulated encounters, focusing on usability, accuracy, and communication access.

## Methods

### Background

The pilot took place in a patient room at one of the Department of Family Medicine clinics. The primary goal was to assess the feasibility and acceptability of a real-time captioning tool in a clinical setting. Secondary objectives included evaluating ease of use, distraction, trust, and satisfaction, factors critical to determining whether the tool supports communication access. Quantitative and qualitative data were collected concurrently within the same study session using a convergent parallel mixed-methods design. Participants completed postscenario surveys and a brief structured exit interview during the same visit, allowing us to analyze both datasets in parallel before merging findings during the interpretation phase.

### Recruitment

We recruited participants who self-identified as DHH through internal email lists compiled from prior studies, social media, and snowball sampling. Inclusion criteria included people who were DHH, preferred to communicate in spoken English, and were at least 18 years old. Recruitment materials explained that the study evaluated a real-time captioning system in simulated medical scenarios.

### Mock Clinical Scenarios

**Figure 1.**
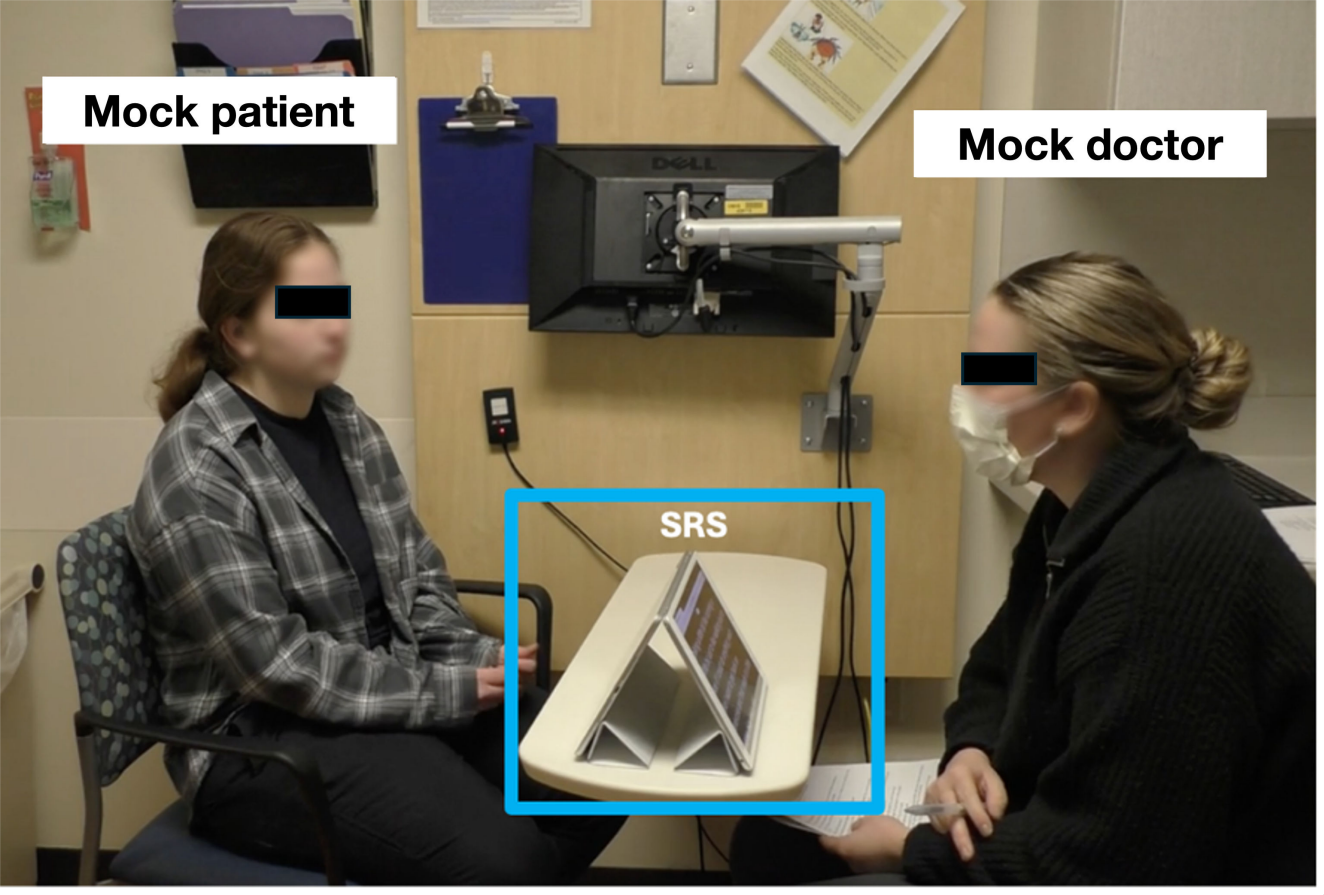
An example of a mock clinical scenario with the real-time speech recognition system set up on a table between the participant (left) and the mock doctor (right). A microphone on the iPad facing the mock doctor detects audio during interviews. Transcripts are displayed on both iPads in real time. SRS: speech recognition system.

Participants completed 3 mock clinical scenarios using the automated speech recognition system (SRS) which was developed by us. The SRS used Google’s speech-to-text application programming interface to transcribe speech to text with low latency and competitive accuracy [24]. The setup included 2 iPads arranged in a tented position so that each device faced either the participant or the mock doctor. Both iPads displayed the generated captions simultaneously (Figure 1).

Before each experiment, we used a random number generator to assign scenario order for each participant. Two team members (both medical students) alternated between serving as the mock doctor (administering scenarios) or facilitator (administering postscenario surveys and exit interviews).

The scenarios were based on commonly reported primary care concerns: (1) back pain, (2) headache, and (3) high blood pressure. Scenario scripts were designed by trained medical students and a clinical faculty member to closely replicate real clinical conversations. The mock doctors wore surgical masks to simulate real-life communication barriers, such as muffled sound and loss of visual cues.

### Postscenario SRS Assessments

Following each scenario, participants provided feedback on the captioning system, rating it across 4 domains: distraction, ease of use, trust, and overall satisfaction (Multimedia Appendix 1). Scenario-specific questions included: “In your discussion with the mock doctor, how distracting were the captions?” “How easy or difficult was it to watch the caption while talking with the mock doctor?” “How much did you trust the accuracy of the generated captions?” “In this scenario, how satisfied were you with the captioning technology?” To reduce response bias, we alternated the direction of the scales: ease of use and trust rated from 1 (strong agreement) to 5 (strong disagreement), and satisfaction rated from 1 (strong disagreement) to 5 (strong agreement). Distraction was scaled separately from 1 (strong disagreement) to 3 (strong agreement).

### Participant Survey Questions

To evaluate user experience with the SRS, participants completed a structured exit interview consisting of 9 questions (5 scalar and 4 open-ended items; Multimedia Appendix 1). To ensure accessibility, a study team member read all questions aloud while they were displayed on an iPad (Apple Inc). We audio-recorded and transcribed responses verbatim using a third-party service, then deidentified the transcripts. We reviewed audio files to clarify unclear segments. Given the brief interviews, we organized and analyzed responses in Microsoft Excel (version 16.77).

Open-ended responses were reviewed using a structured framework aligned with predefined domains: ease of use, comfort, satisfaction, trust, emotional response, and the captioning system’s ability to support or replace lip reading. Overall, 3 team members (SEH, LJM, and LW) independently applied initial codes to a subset of transcripts. Coding discrepancies were resolved through discussion, and the codebook was refined iteratively. Consistency was maintained through regular team meetings, and reflexive discussions were used to address potential bias.

Themes were identified based on frequency, relevance to study aims, and salience across participants. Representative participant comments were selected to illustrate key insights. Thematic saturation was reached when no new concepts emerged from successive interviews.

### Mixed Methods Integration

To integrate quantitative and qualitative data, we used a convergent parallel approach in which both datasets were collected during the same phase, analyzed separately, and then merged during the interpretation phase. Integration occurred through (1) narrative weaving of findings across domains and (2) construction of a joint display that juxtaposed quantitative ratings with representative qualitative insights to generate meta-inferences. This approach allowed identification of areas of convergence and divergence between usability ratings and participants’ lived communication experiences.

### Closed Captioning Accuracy

In addition to participant feedback, we analyzed the accuracy of the system’s transcriptions. We compiled all transcripts generated by the mock doctors and compared them to professional communication access real-time translation transcripts.

We used word error rate (WER), a standard metric in automatic speech recognition (ASR) that calculates errors as the ratio of insertions, deletions, and substitutions required to align the system output with the reference [25,26]. We implemented WER calculations using the Python-based *jiwer* library, which provides standardized scoring for automated SRSs. This approach allowed us to assess how closely the SRS-generated captions matched professional-level transcription, validating the system’s effectiveness in realistic use cases.

### Statistical Analysis

We performed univariate analyses on demographic data and postscenario survey responses. Because of the small sample size, the study was not powered to detect subgroup differences. For transcript analysis, we segmented transcripts from mock sessions into 3 distinct scenarios. To focus on the primary use case, captioning clinician speech, we excluded utterances from participants who are DHH and analyzed only the mock doctors’ speech.

### Ethical Considerations

This study was approved by the University of Michigan Institutional Review Board (IRB; HUM00240244). All participants provided informed consent prior to participation. Participants were informed of the study purpose, procedures, potential risks, and their right to withdraw at any time without penalty. All study data were deidentified prior to analysis, and transcripts were reviewed to remove personally identifiable information. Audio recordings and transcripts were stored on secure, password-protected institutional servers accessible only to the study team. Participants received a US $25 Amazon gift card for their participation. The individuals depicted in the figure provided explicit written consent for publication of their images. The individuals shown in Figure 1 provided explicit written consent for their images to be published.

## Results

### Participant Characteristics

Overall, 11 participants who are DHH enrolled and participated in the pilot study. Due to equipment failure resulting in complete data recording loss with Participant 5, this participant was excluded from the analysis. The 10 remaining participants had an even distribution of genders (Table 1).

**Table 1.** Study participant demographics.

ID	Age (years)	Sex	Identity	Hearing loss levels^a^	Wearable technology^b^	Lip reader
P01	61	Female	HoH^c^	Severe	Yes	All of the time
P02	66	Male	HoH	Severe	Yes	Sometimes
P03	66	Male	HoH	Severe	Yes	No
P04	66	Female	HoH	Moderately severe	Yes	Sometimes
P06	43	Female	Deaf	Profound	Yes	All of the time
P07	21	Female	deaf	Moderately severe	Yes	Sometimes
P08	39	Female	HoH	Severe	Yes	All of the time
P09	24	Male	Deaf	Profound	Yes	No
P10	56	Male	deaf	Profound	Yes	Sometimes
P11	20	Male	HoH	Mild	Yes	Sometimes

a Hearing loss levels were self-identified, and all participants reported equal hearing loss levels bilaterally.

b Wearable technology includes hearing aids and cochlear implants.

c HoH: hard of hearing.

The mean age of the participants was 46.2 (SD 19.3) years. Six participants identified as “hard of hearing,” 22 as “Deaf,” and 2 as “deaf.” Seven participants self-reported severe to profound HL, and all participants had bilateral HL. Five participants self-reported congenital HL, 2 reported childhood onsets of HL (<12 y old), and 2 reported HL as adults (>18 y old). Hearing aids were used by 8 participants, and 2 participants used cochlear implants. Seven participants used captioning services in the past. Seven also incorporated smartphone-based hearing assistive technology. Three used “other” tools, including using cupped hands behind ears to assist in hearing. Eight participants reported varying degrees of dependence on lip reading, but 5 participants depended sometimes on lip reading and 5 depended fully on lip reading.

### Postscenario SRS Assessments

There were 29 postscenario SRS assessment surveys, 3 survey responses each from 9 participants and 2 survey responses from 1 participant. One survey response from participant P11 was not collected due to a technician error. Overall, participants found the captioning technology not distracting in 86% (25/29) of scenarios (Table 2). In 90% (26/29) of scenarios, participants trusted the accuracy of generated transcription and felt the captions were easy to watch while conversing with the mock doctor. In 76% (22/29) of scenarios, participants were satisfied with the captioning technology. The technology was least satisfying to participants in the back pain scenarios (70% satisfaction) compared to the high blood pressure (78% satisfaction) and headache (80% satisfaction) scenarios.

**Table 2.** Summary of participant assessments regarding live captioning technology compiled from all 3 scenarios and dichotomized.

Questions^a^	Assessments	Values, n (%)	
In your discussion with the mock doctor, how distracting were the captions?	Not distracting^b^	25 (86)	
How easy or difficult was it to watch the caption while talking with the mock doctor?	Easy^c^	26 (90)	
How much did you trust the accuracy of the generated captions?	Trusted^d^	26 (90)	
In this scenario, how satisfied were you with the captioning technology?	Satisfied^e^	22 (76)	

a For all 4 questions, n=29 since 1 of the 10 participants did not participate in 1 of the 3 scenarios.

b Not distracting: not at all distracting.

c Easy: very easy + somewhat easy.

d Trusted: completely trusted + somewhat trusted.

e Satisfied: very satisfied + somewhat satisfied.

### Participant Experience Surveys

All 10 participants completed structured exit interviews following the captioning scenarios, providing reflections on their overall experience with the SRS (Table 3). Interview responses were analyzed using a predefined framework aligned with domains explored in the postscenario ratings (eg, ease of use, comfort, satisfaction, trust, emotional impact, and support for lip reading). This section summarizes participant perspectives and provides representative quotes to contextualize the quantitative results described above.

**Table 3.** Representative participant reflections by theme.

Themes	Relevant quotes^a^	Interpretation
Ease of use	“At first I wasn’t sure what to expect, but after a few lines of text I stopped even thinking about it—it just worked. That made me feel more in control.” (P04)	Participants found the system intuitive and accessible.
Comfort	“I didn’t have to strain or overthink. It just flowed naturally and I didn’t even realize how relaxed I was until the end.” (P08)	Technology reduced cognitive effort and fostered emotional ease.
Satisfaction	“I was happy. I wish all the doctors would have something like this. It made me feel like my experience mattered.” (P03)	Participant expresses satisfaction and a sense of being valued.
Safety and trust	“Because it’s live, it feels very safe. You’re not left guessing, and I felt confident nothing important was missed.” (P01)	Real-time functionality enhanced user confidence and perception of safety.
Emotional response	“I didn’t realize how much stress I usually carry during appointments. This made me feel heard and like I could finally breathe.” (P09)	System reduced communication-related anxiety and supported emotional well-being.
Support or replace lip reading	“With the mask on, it would have been extremely difficult to follow—and with the captioning, it was just leaps better. I wasn’t exhausted from trying to read lips the whole time.” (P07)	The technology was viewed as a vital alternative to lip reading, especially in masked settings.

a Relevant quotes from individual participants illustrating each core theme, including insight into perceived usability, comfort, satisfaction, emotional impact, and the role of real-time captions in supporting communication.

Most participants (9/10) described the system as easy to use, frequently using phrases like “very easy” or “easier than usual.”

One participant remarked,

*After a few lines of text I stopped even thinking about it—it just worked. That made me feel more in control.*

Another noted,

*It was easier than usual because we don’t have captioning. It’s always nice to have it just in case you miss something.*

Participants also reported high comfort with the system. Descriptions included “very comfortable,” “easy to work with,” and “high comfortability.*”*

As 1 participant shared,

*It just flowed naturally, and I didn’t even realize how relaxed I was until the end.*

Satisfaction was also high across interviews. While 76% of scenario ratings reflected satisfaction, all participants described themselves as satisfied or very satisfied in exit interviews. One stated,

*I was happy. I wish all the doctors would have something like this.”*

Another shared,

*I was pretty satisfied, and the captioning was spot-on.*

When asked about trust in the system, participants frequently described the captions as reliable. One participant reflected,

*Because it’s live, it feels very safe. You’re not left guessing.*

A few raised questions about data privacy, with one noting,

*I would also want to know what happens to the transcript and who has access to it.*

Participants also described emotional benefits from the technology. In total, 9 of 10 participants used words like “reassured,” “relaxed,” and “comfortable” to describe how the SR*S* made them feel. One participant shared,

*This made me feel heard and like I could finally breathe.*

Perceptions of the captions’ ability to support or replace lip reading were more varied. Several participants described the system as a helpful supplement or improvement, particularly in masked settings. As one noted,

*With the mask on, I definitely depended on it more.*

Another stated,

*I think it’s better than lip reading.*

Others expressed that lip reading remained important, with one participant saying,

*Not going to replace lip reading... captions help, but I still rely on visual cues.*

Beyond these predefined domains, participants spontaneously shared reflections on broader applications of the SRS. Several expressed enthusiasm for expanding its use in real-world clinical settings, with one stating,

*I would like to see that in many doctor’s offices tomorrow.*

Others suggested the system may be particularly helpful for patients who are older, have cognitive impairments, or use interpreters. A few noted that having real-time captions reduced the pressure to maintain constant visual attention, allowing for more natural communication and less fatigue.

### Closed Captioning Accuracy

We collected and preprocessed transcripts from 10 mock clinical sessions. Due to varying levels of verbosity among the participants, the total transcript lengths varied substantially, ranging from 1144 to 4704 words.

Overall, participants found the SRS to be sufficiently accurate (Table 4). For instance, P04 noted that the system was *“more accurate than the phone captions”* she typically uses in daily conversations. Similarly, P06 commented on the system’s effectiveness compared to human captioners, stating,

*A lot of the captions I had were court reporters*—*they caption fast, but sometimes they make mistakes. ... And this one [the SRS], it’s more accurate and I see words better.*

Nonetheless, participants expressed concerns about the system’s ability to handle more complex or specialized medical vocabulary. For example, P10 questioned *“how it would be with more complex medical terminologies,”* in real clinical settings where more technical jargon and medication names were frequently used.

**Table 4.** The word error rate for each scenario, along with the accumulated word error rate for each participant across all 3 scenarios. These word error rate scores specifically reflect the accuracy of the automated speech recognition system in transcribing the mock doctors’ speech.

ID	Mock doctor	Scenario 1^a^	Scenario 2^a^	Scenario 3^a^	Accumulated (range: 0.127-0.167)
P01	M1	0.136	0.133	0.125	0.131
P02	M2	0.193	0.141	0.133	0.153
P03	M1	0.129	0.122	0.133	0.127
P04	M2	0.228	0.128	0.151	0.167
P06	M2	0.137	0.152	0.135	0.144
P07	M1	0.127	0.134	0.132	0.133
P08	M2	0.155	0.142	0.152	0.149
P09	M1	0.136	0.131	0.141	0.137
P10	M1	0.127	0.126	0.133	0.129
P11	M2	0.147	0.185	0.134	0.151

a The scenario-level word error rates ranged between 0.122 and 0.228.

### Joint Display of Integrated Findings

To illustrate convergence between quantitative usability ratings and qualitative interview themes, we constructed a joint display summarizing merged findings and resulting meta-inferences across key domains (Table 5).

**Table 5.** Joint display of integrated quantitative and qualitative findings

Domain	Quantitative result	Representative quote	Integrated meta-inference
Ease of use	90% rated captions “easy”	“After a few lines of text I stopped even thinking about it—it just worked.”	High usability with minimal cognitive load Captions supported natural conversational flow
Comfort	Not directly measured	“It just flowed naturally, and I didn’t realize how relaxed I was.”	Technology reduced strain and fostered emotional ease during communication
Satisfaction	76% satisfied	“I wish all the doctors would have something like this.”	Satisfaction tied to both functional value and feeling understood and supported
Safety and trust	90% trusted accuracy	“Because it’s live, it feels very safe. You’re not left guessing.”	Real-time display strengthened perceived safety and reliability despite minor errors
Emotional response	Not directly measured	“This made me feel heard and like I could finally breathe.”	Captions enhanced psychological safety and reduced anxiety—benefits not captured numerically
Support or replace lip reading	Not directly measured	“With the mask on, I depended on it more… it was leaps better.”	Captions supplemented or replaced lip reading, reducing fatigue in masked settings

## Discussion

### Principal Results

To successfully deploy SRS in clinical settings, it is essential that the system accurately captures and reflects clinicians’ speech. Our findings show that although the SRS output was not flawless, its WERs fell between 0.10 and 0.20, a range generally considered acceptable for real-world ASR use [19,26]. Furthermore, participants understood the captions with relative ease, suggesting that transcription quality was sufficient to support comprehension in simulated outpatient scenarios. However, stricter accuracy standards may be required in high-stakes contexts, such as discussions of medications or treatment options, where small errors can have serious consequences.

Although WER is widely used to evaluate ASR performance, it weighs all error types equally, regardless of their impact on comprehension [27]. Prior work has proposed alternative evaluation approaches that aim to capture semantic accuracy or user-centric measures of intelligibility and usefulness [20]. In clinical communication, we support developing evaluation metrics that align more closely with safety-critical requirements. Such metrics would be instrumental in determining when ASR systems are truly ready for deployment in health care environments. In clinical settings, misrecognition of medical terminology can have consequences far more serious than common transcription errors, especially when involving medication names, diagnoses, or treatment instructions. Because of this, future work should consider safety-critical evaluation frameworks that go beyond traditional WER. Approaches, such as semantic error analysis, comprehension-based scoring, or accuracy, weighting for medically significant terms could better capture the real-world implications of captioning errors in health care communication.

Our participants represented a variety of ages, genders, HL levels, and degrees of dependence on lip reading. However, most participants had previously used captioning technology as an accommodation, so our usability findings may be less generalizable to individuals who are DHH with no prior captioning experience. Also, only 2 participants preferred written communication with hearing people. Therefore, satisfaction with our captioning technology may be higher than our results suggest for people who are DHH and depend more on written communication. Regardless of the scenario, most participants were satisfied with the SRS, trusted its accuracy, found it easy to watch, and were not distracted.

Participants trusted the captioning system despite occasional transcription errors, which embodies the concept of trust-in-automation frameworks, where user reliance is shaped by perceived system reliability and predictability [28]. Exit interviews revealed that beyond meeting technical expectations, the captioning system also meaningfully supported emotional connection, trust, and autonomy during clinical interactions. Participants described the captions as easy to use and grounding. They also noted reduced stress, lower cognitive fatigue, better understanding, and a stronger sense of being heard. Encouragingly, the observed reduction in stress and fatigue is consistent with prior work where assistive technology helped manage cognitive effort during information processing [29,30]. These findings suggest that accessibility tools should be evaluated not only by their accuracy but by their ability to support psychological safety and communication equity [31].

Additionally, although participants generally trusted the captioning system, a few raised concerns about transcript privacy and data handling. These concerns highlight the ethical need for transparency when implementing automated captioning in health care. This pilot used secure, locally stored recordings without identifiable data, but clinical deployment will require Health Insurance Portability and Accountability Act (HIPAA)-compliant encryption and explicit consent protocols. Adding user controls, such as options to delete transcripts or disable storage, could further strengthen trust among users who are DHH and other vulnerable populations. Nevertheless, participants recommended broader adoption of SRS, particularly for older adults and others facing progressive hearing-related communication barriers, underscoring the system’s potential to improve care for a heterogeneous population of DHH patients.

### Limitations

While our findings are promising, this study has several limitations. Most participants were experienced caption users and had prior familiarity with assistive communication technologies, which may have positively influenced usability and satisfaction ratings. As a result, these findings may not fully represent the experiences of individuals who are DHH and are less familiar with captioning or other accessibility tools or primary American Sign Language users. Future research should include participants with varying levels of captioning experience and a broader demographic range to better assess generalizability and identify barriers for first-time users. This study was conducted in controlled, simulated settings, which may not fully reflect the complexity and spontaneity of real-world medical encounters. Because these mock scenarios involved medical students rather than practicing clinicians, the communication dynamics may differ from authentic physician–patient interactions. Future work should therefore include real-world clinical deployments to evaluate how captioning systems perform in active care settings and adapt to diverse communication styles and environmental conditions.

Second, although our participant pool included individuals with diverse hearing identities and varying degrees of familiarity with assistive technologies, it does not capture the full range of experiences within the broader community who are DHH. Future work should include longitudinal application in various clinical settings and recruitment of a more diverse participant population to better assess long-term usability and impact.

In addition, our SRS was not specifically optimized for medical vocabulary. This limitation was evident in the system’s tendency to misrecognize medical terminology, words that are infrequent in everyday speech yet crucial for accurate clinical communication. Furthermore, while we used WER as a standard quantitative evaluation metric, it does not fully capture how users who are DHH interpret and understand captions, particularly in high-stakes contexts. Future research should explore the development of domain-specific SRS trained on medical speech and adopt evaluation metrics that better reflect comprehension and user experience among individuals who are DHH. Finally, since WER assigns equal weight to all tokens, it does not differentiate between routine transcription errors and those involving safety-critical clinical terms (eg, medications or diagnoses). Therefore, WER may underestimate the potential impact of certain errors in medical contexts.

### Future Directions

Improving SRS accuracy for medical terminology remains a key technical priority for clinical use. Strategies may include (1) speech recognition models on deidentified clinical audio to capture the acoustic variability of real-world medical speech [32], (2) embedding domain-specific medical dictionaries and medication name libraries into the language model of the SRS systems to reduce substitution errors [33], (3) leveraging context-aware large language models that can infer meaning from partial or uncertain input [34], and (4) integrating clinician feedback loops for rapid correction of recurring misinterpretations [35]. These enhancements would not only improve accuracy for technical vocabulary but also strengthen user trust and perceived reliability in clinical environments.

Building on these preliminary findings, future work should also explore integration with medical-domain ASR models to enhance accuracy for specialized terminology and complex clinical dialog. Longitudinal studies will be valuable for assessing maintained usability, user trust, and performance over time. Additionally, testing captioning systems in broader clinical contexts, such as emergency care, geriatrics, and among patients with cognitive impairment, will help determine their adaptability and impact across diverse care settings.

### Implications for Clinical Workflow Integration

Our findings demonstrate that real-time captioning is usable and beneficial in clinical settings for patients who are DHH, aligning with prior evidence that captioning improved recall of anesthesia-related consent conversations [36]. Given this demonstrated value, practical integration of captioning tools into clinical workflows will require thoughtful design to minimize disruption while enhancing accessibility. Participants envisioned use cases in which SRS displays could be embedded within existing electronic health record systems or mirrored on clinician tablets to preserve natural eye contact and conversational flow. Integration will also depend on clear institutional protocols for activating captioning on demand, ensuring confidentiality, and providing clinician training on how to engage with patients who are DHH using this technology. Establishing these processes could enable captioning to function as a routine accessibility feature rather than an exception, supporting both efficiency and equitable communication in care delivery.

### Conclusions

This pilot study demonstrates that artificial intelligence–enhanced captioning can meaningfully improve communication experiences for individuals who are DHH in clinical settings. Participants found the system intuitive, emotionally supportive, and effective in bridging common communication barriers, especially those worsened by face masks and unfamiliar environments. While traditional captioning tools often fall short in medical contexts, integrating large language models into the speech recognition process offers a promising path toward more coherent, accurate, and human-centered accessibility. By centering on user perspectives, this study highlights the importance of evaluating assistive technologies not only for transcription quality, but for their impact on trust, inclusion, and psychological safety. Future research should build on these early insights to further refine captioning systems, examine their use in real-world clinical care, and ensure that patients who are DHH are active partners in the design of accessible digital health solutions.

## Supplementary material

10.2196/79073 Multimedia Appendix 1 Structured exit interview questions.

## Abbreviations

ASR: automatic speech recognition
DHH: Deaf/deaf or hard of hearing
HIPAA: Health Insurance Portability and Accountability Act
HL: hearing loss
SRS: speech recognition system
WER: word error rate
