# Supplementary material for: Assessing the Role of Medical Caption Technology to Support Physician-Patient Communication for Patients With Hearing Loss: Mixed Methods Pilot Study
Source: JMIR Rehabil Assist Technol. 2026 Jan 15;13:e79073. doi: 10.2196/79073 (PMC12806592; doi:10.2196/79073)
Supplement: Multimedia Appendix 1 [file rehab-v13-e79073-s001.docx]

**Table S1.** Participants were asked the following structured questions during the exit interview, administered both verbally and visually. Interviewers were allowed to ask follow-up prompts for clarification or elaboration.

| **Domain** | **Question** |
| --- | --- |
| Ease of Use | 1. Given our captioning technology, how easy or difficult was it to communicate during these scenarios? |
| Comfort | 1. How comfortable were you using our captioning technology? |
| Satisfaction | 1. How satisfied were you with our captioning technology? |
| Comparison | 1. If you have prior experiences with live captioning, how does our captioning technology compare to other live captioning technology you have used? |
| Safety and Trust | 1. How safe or unsafe would you feel about using our technology in a medical setting? |
| Emotional Response | 1. What words would you use to describe how you felt using this technology? |
| Support for Lip Reading | 1. To what extent do you think captions like these could replace lip reading? |
| General Reflections | 1. What other comments do you have about this experience as a whole? |
